# Inhibitory effect of *Sanguisorba hakusanensis* Makino ethanol extract on atopic dermatitis-like responses in *NC/Nga* mice and human keratinocytes

**DOI:** 10.1038/s41598-023-41676-3

**Published:** 2023-09-05

**Authors:** Hyun-Kyung Song, Sun Haeng Park, Hye Jin Kim, Seol Jang, Byung-Kil Choo, Ho Kyoung Kim, Taesoo Kim

**Affiliations:** 1 KM Convergence Research Division, Korea Institute of Oriental Medicine, Yuseong-daero 1672, Yuseong-gu, Daejeon, 34054 Republic of Korea; 2 College of Pharmacy, Seoul National University, 1 Gwanak-ro, Gwanak-gu, Seoul, 08826 Republic of Korea; 3 Department of Crop Science and Biotechnology, Jeonbuk National University, Jeonju, 54896 Republic of Korea

**Keywords:** Chemokines, Inflammation, Cell signalling

## Abstract

Atopic dermatitis (AD) is an allergic, inflammatory skin disease caused by immune dysregulation. In this study, we investigated anti-atopic and anti-inflammatory activities of *Sanguisorba hakusanensis* ethanol extract (SHE) both in vivo using *NC/Nga* mice and in vitro using human HaCaT keratinocytes. Oral administration of SHE suppressed several atopic symptoms associated with house dust mites (induced with *Dermatophagoides farinae* extract) in *NC/Nga* mice and decreased serum levels of inflammatory mediators such as immunoglobulin E, histamine, and inflammatory chemokines. Additionally, SHE treatment reduced the infiltration of immune cells such as mast cells and macrophages in AD skin lesions. In vitro, interferon-γ- and tumor necrosis factor-α-stimulated HaCaT cells exhibited increased expression of T helper 1 and 2 chemokines; their expression was inhibited by SHE treatment. The anti-inflammatory effects of SHE treatment involved blocking of the mitogen-activated protein kinase and signal transducer and activator of transcription 1 signaling pathways. In conclusion, SHE exerts potent anti-atopic and anti-inflammatory effects and should be considered for the clinical treatment of AD.

## Introduction

Atopic dermatitis (AD), a chronic inflammatory skin disease caused by an imbalance in the immune response to allergens, is mainly observed in infants as well as children and is highly likely to recur^1^. The common symptoms of AD are skin dryness, thickening, inflammation, hypersensitivity, and itching^2^. Patients with AD exhibiting these symptoms have high levels of several inflammatory mediators such as immunoglobulin E (IgE), histamine, as well as inflammatory cytokines and chemokines^2,3^. These inflammatory mediators are secreted by various immune cells, including lymphocytes, macrophages, eosinophils, and mast cells, which infiltrate AD skin lesions^4,5^.

*NC/Nga* mice are an ideal animal model for studying allergen-induced AD because they exhibit etiology and clinical symptoms similar to those in humans with AD^6–8^. In addition, chronic itching inevitably leads to scratching behavior, with the continuation of the itching–scratching cycle further aggravating atopic inflammation and skin barrier dysfunction^9^. House dust mites are the most common cause of AD. *Dermatophagoides farinae* is a major species of house dust mite. Its body and feces, which are known environmental allergens, cause atopic skin inflammation in *NC/Nga* mice^10–12^. In addition, activated keratinocytes play a crucial role in the pathogenesis of AD. Interferon (IFN)-γ and tumor necrosis factor (TNF)-α stimulate epidermal keratinocytes to induce the release of inflammatory chemokines^13–15^.

Although corticosteroids and antihistamines have been widely used in AD treatment, studies suggest that their continued use can cause serious side effects^16^. Therefore, the development of safer and effective therapeutics from natural sources is necessary for efficient AD treatment. *Sanguisorba*, commonly known as burnet, is a genus in the family Rosaceae and is native to the temperate regions of the northern hemisphere. Six *Sanguisorba* species, commonly called “Zi-Yu,” are distributed throughout Korea. These species have been used as traditional herbs in China and Korea^17,18^. Traditionally, they have been used alone or in combination with other herbs to effectively treat all types of hemorrhage, diarrhea, chronic intestinal infections, and duodenal ulcers^18,19^. Among the *Sanguisorba* species*, S. officinalis* possesses various pharmacological properties, including anti-inflammatory^20^, anti-allergic^21–24^, anti-cancer^25,26^, anti-bacterial^27^, and anti-obesity properties^28,29^. Although *S. officinalis* has been extensively studied relative to other *Sanguisorba* native to Korea, only few studies on *S. hakusanensis* Makino (SH) exist. Further, none of the studies have attempted to determine the anti-atopic properties of SH. In this study, we investigated the anti-atopic activities of SH ethanol extract (SHE) in *D. farinae* extract (DFE)-treated *NC/Nga* mice and IFN-γ/TNF-α-stimulated human HaCaT keratinocytes.

## Results and discussion

### Inhibitory effects of SHE on DFE-induced AD-like symptoms and scratching behavior in NC/Nga mice

**Figure 1:**
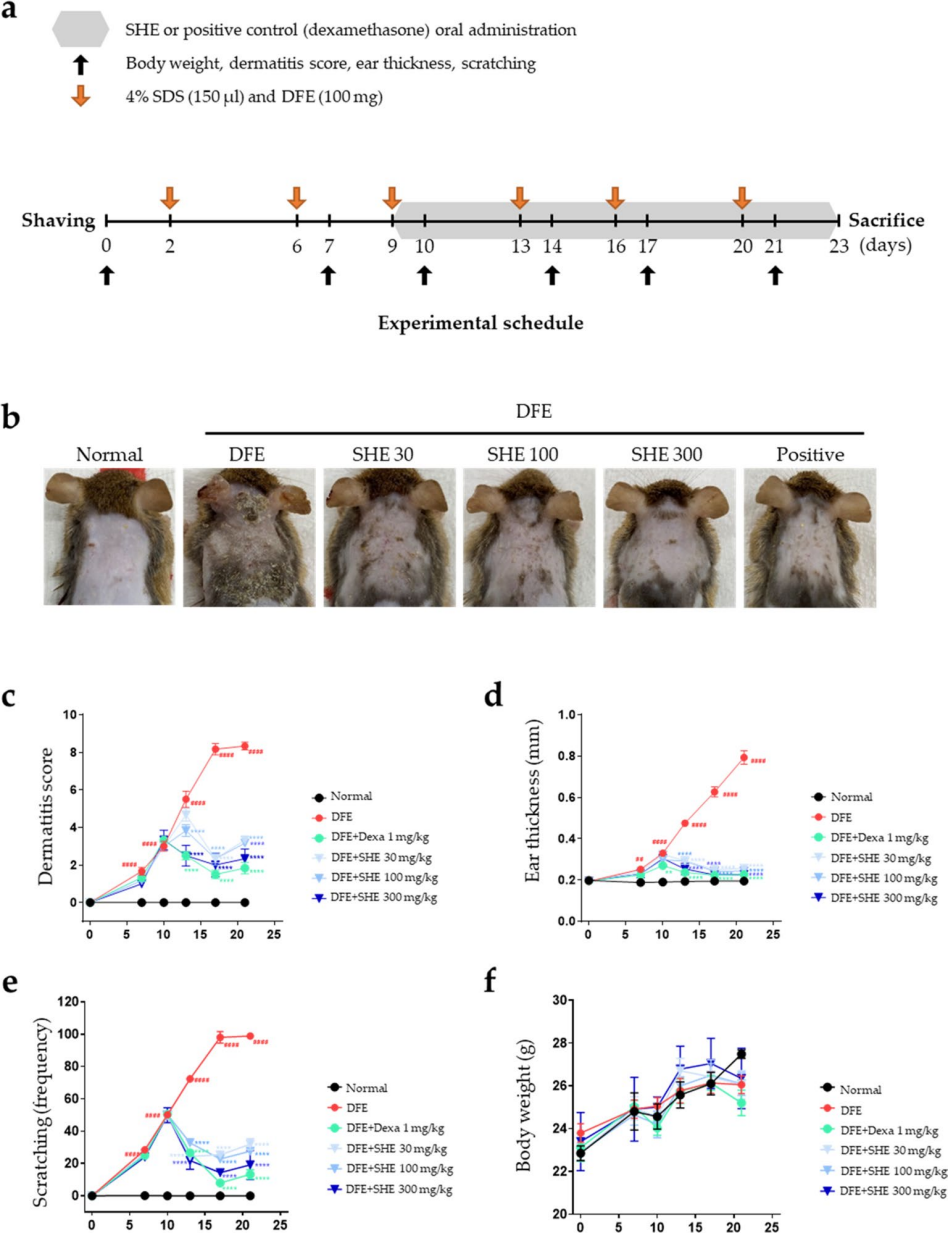
*Sanguisorba hakusanensis* ethanol extract (SHE) alleviates *Dermatophagoides farinae* extract (DFE)-induced AD-like skin lesions in *NC/Nga* mice. (**a**) Experimental scheme for investigating the effects of SHE in an NC/Nga mouse model with DFE-induced AD. (**b**) Photographs of mouse dorsal skin and ear lesions from each group on the last experimental day. (**c**) Dermatitis score, (**d**) ear thickness, (**e**) scratching behavior, and (**f**) body weight were evaluated twice a week for 3 weeks. Values are presented as mean ± SEM (n = 6). #*P* < 0.05, ##*P* < 0.005, ###*P* < 0.0005, ####*P* < 0.0001 vs. the normal group, *P < 0.05, **P < 0.005, ***P < 0.0005, ****P < 0.0001 vs. the DFE group.

To investigate the effects of SHE on AD skin lesions, DFE was administered twice a week on the shaved dorsal skin and ear of *NC/Nga* mice for 3 weeks (Fig. 1a). Repeated dorsal skin and ear local application of DFE significantly aggravated AD symptoms such as swelling, erythema, cornification, exudation, dry skin, and ear thickness. Subsequent treatment with SHE significantly relieved AD-like symptoms, such as ear thickness and dermatitis score, in DFE-treated *NC/Nga* mice (Fig. 1b–d). Allergens such as DFE cause itching and scratching behavior that contributes to the over-activation of the immune response^3,30^. The scratching behavior of mice was visually observed, and the frequency of scratching in a 20-min period was determined. Compared with DFE treatment, treatment with SHE and dexamethasone suppressed the scratching behavior in *NC/Nga* mice (Fig. 1e). In addition, daily oral administration of SHE for 3 weeks did not significantly change mouse weight compared with the DFE treatment (Fig. 1f), indicating that SHE did not exert a toxic effect. These results suggest that SHE treatment alleviates AD-like symptoms of skin and ear lesions in DFE-treated *NC/Nga* mice.

### Inhibitory effects of SHE on the DFE-induced histological features and immune cell infiltration in NC/Nga mouse skin lesions

**Figure 2:**
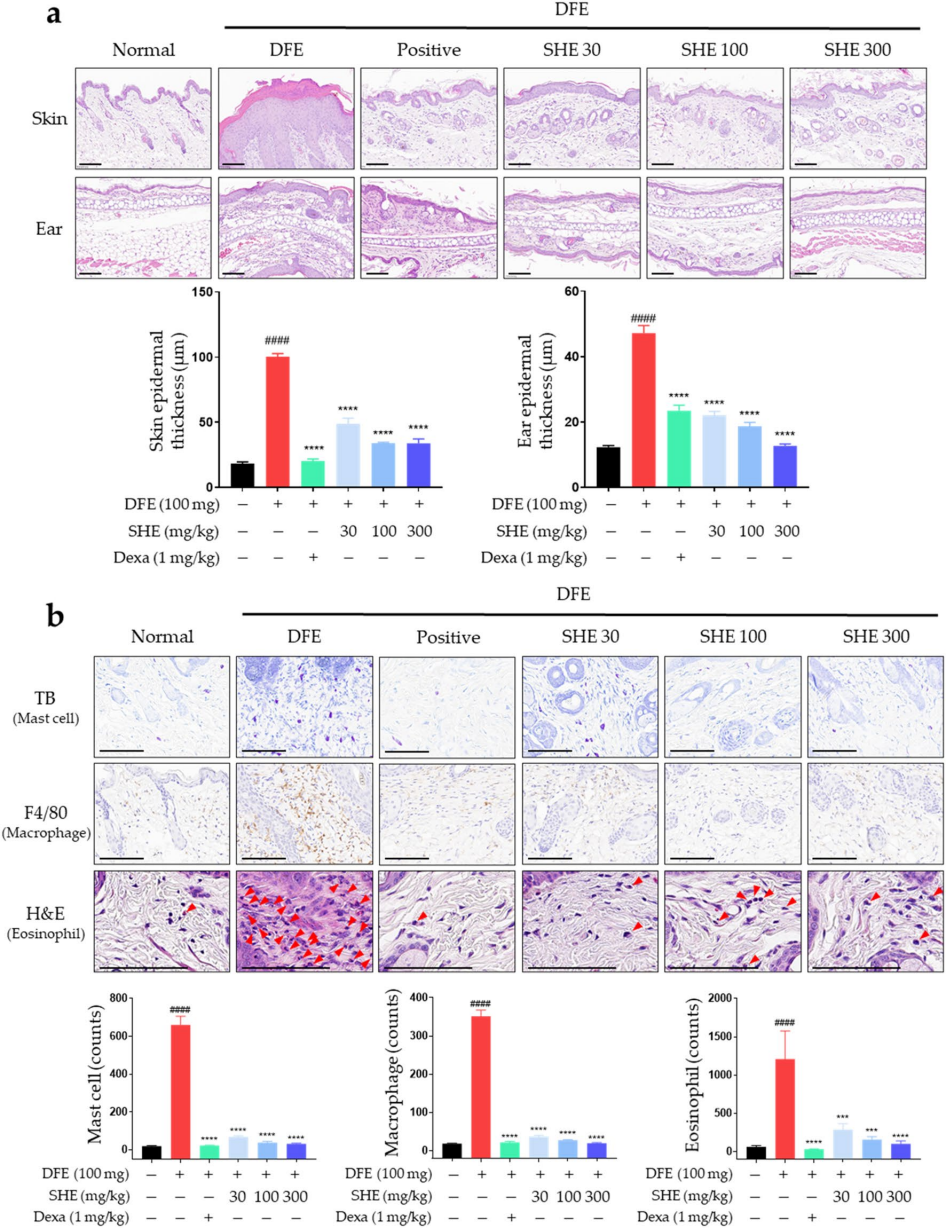
Effect of *Sanguisorba hakusanensis* ethanol extract (SHE) on the epidermal thickness and mast cell infiltration in atopic dermatitis (AD)-like skin and ear lesions of *NC/Nga* mice. Sectioned dorsal skin and ears stained with (**a**) hematoxylin & eosin (H&E). The stained sections were observed under a microscope at 400 × magnification. Scale bar = 100 μm. (**b**) Dorsal skin tissue sections were stained with toluidine blue (TB), anti-F4/80 antibodies, and H&E to confirm immune cell infiltration. The stained sections were examined under a microscope at 800 × or 1600 × magnification. Scale bar = 100 μm. Skin and ear epidermal thickness and immune cell infiltration of DFE-treated NC/Nga mice were analyzed; the results are represented as graphs. Values are presented as mean ± SEM (n = 6). #*P* < 0.05 vs. the normal group; *P < 0.05, **P < 0.005, ***P < 0.0005, ****P < 0.0001 vs. the DFE group.

An increase in epidermal thickness is an AD symptom caused by pathologically activated epidermal proliferation via keratinocyte differentiation in inflammatory skin lesions^31^. Therefore, the dorsal skin and ear tissues of *NC/Nga* mice were stained with H&E to determine whether SHE treatment reduces AD-like epidermal thickening. The AD group exhibited increased epidermal thickness compared with the normal group, whereas SHE administration suppressed this increase in a dose-dependent manner (Fig. 2a).

The infiltration of immune cells, such as mast cells and macrophages, in AD eczema lesions is a representative AD symptom. In particular, mast cells infiltrate AD skin lesions and stimulate increased production of IgE and histamine^32^. Eosinophils, integral components of the immune system that collaborate with mast cells, contribute to triggering allergic reactions^33^. Mast cells and eosinophils are attracted to the inflamed dermis, where they are activated, playing a substantial role in exacerbating allergic skin conditions^34^. Studies have revealed the involvement of these cells in modulating itchiness in AD and triggering allergic inflammation^35,36^. Furthermore, mast cells reportedly increase sensory nerve density in the epidermis and dermis^37^. Macrophages play a crucial role in the immune system and host defense during the acute inflammatory phase via inflammatory cytokine release^22,23^ and are associated with the development of various chronic inflammatory diseases, including AD^38^. The F4/80 antigen is a surface glycoprotein frequently expressed on macrophages^39^. In the present study, we determined the numbers of mast cells, macrophages, and eosinophils in the skin lesions of AD in DFE-treated *NC/Nga* mice. This was achieved through the utilization of toluidine blue staining, F4/80 immunohistochemistry, and H&E staining. The administration of SHE inhibited immune cell infiltration in AD-like skin lesions (Fig. 2b). Thus, oral administration of SHE inhibited the increase epidermal thickness and immune cell infiltration in the skin lesions of DFE-treated *NC/Nga* mice.

### Inhibitory effects of SHE on DFE-induced inflammatory mediator production in NC/Nga mice

**Figure 3:**
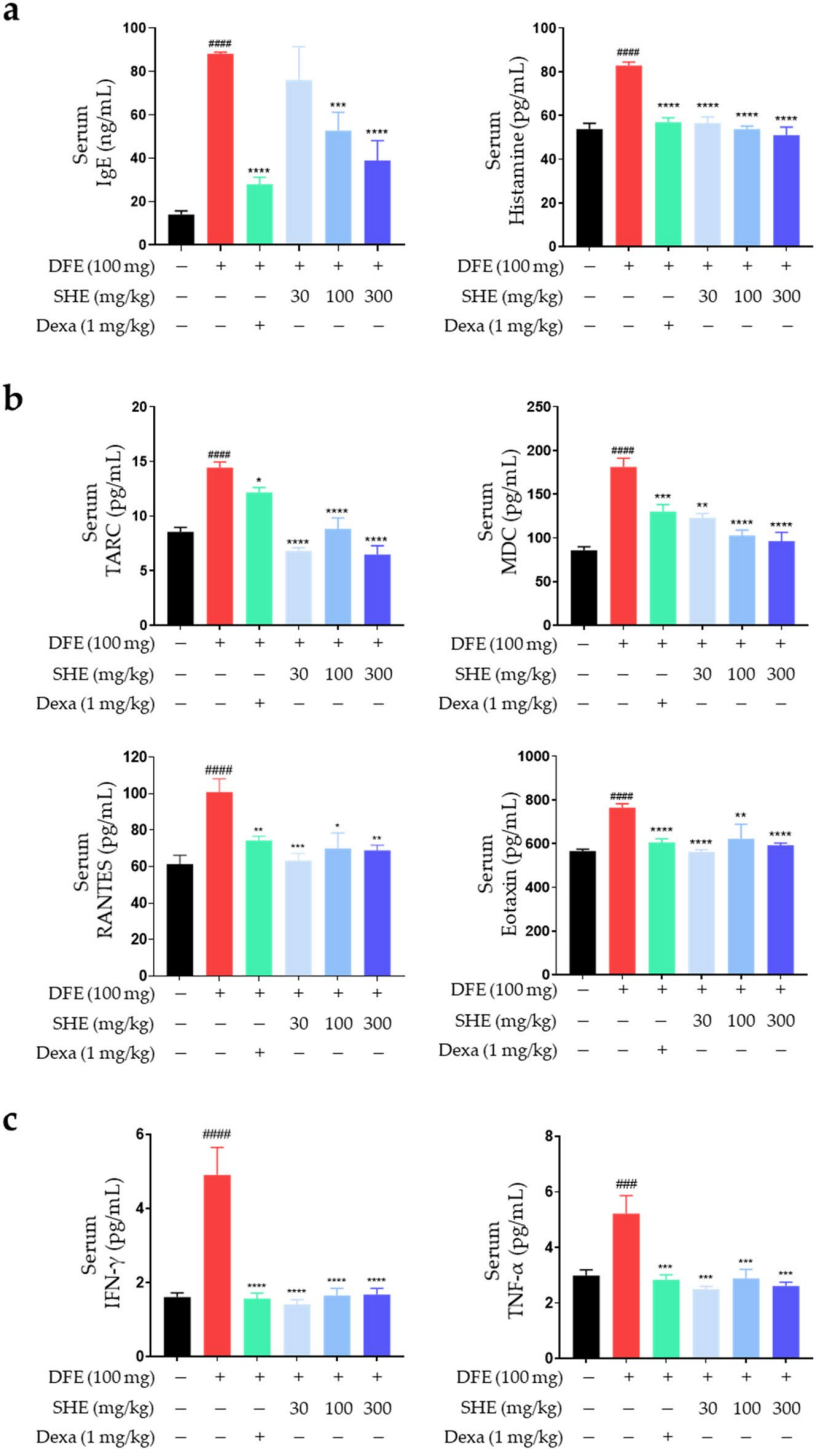
SHE inhibits the release of immunoglobulin E (IgE), histamine, chemokines and cytokines in DFE-induced atopic dermatitis (AD)-like skin lesions in *NC/Nga* mouse serum. (**a**) Serum samples were diluted 100 × times for the experiments. Total IgE and histamine levels in the serum were analyzed using ELISA. (**b**, **c**) Inflammatory chemokine and cytokine levels (TARC, RANTES, MDC, eotaxin, IFN-γ and TNF-α) were confirmed using a bead-based immunoassay. Results are presented as mean ± SEM (n = 6). (c) Inflammatory cytokine levels (IFN-γ and TNF-α) were ^#^*P* < 0.05, ^##^*P* < 0.005, ^###^*P* < 0.0005, ^####^*P* < 0.0001 vs. the normal group; *P < 0.05, **P < 0.005, ***P < 0.0005, ****P < 0.0001 vs. the DFE group.

Allergic inflammatory reactions, including AD, are mainly characterized by an imbalance in various types of T helper (Th) cells during the immune response^40^. IgE is related to the Th2 immune response, which leads to the release of various inflammatory mediators, including histamine and cytokines, through the activation of mast cells^32^. The activated mast cells degranulate, secreting proinflammatory cytokines and histamine, which exacerbate AD-related itching^41^. Therefore, the inhibitory effect of SHE on the release of IgE and histamine in DFE-induced *NC/Nga* mouse serum was determined using ELISA. SHE treatment inhibited the increase in serum IgE and histamine levels in DFE-treated *NC/Nga* mice in a dose-dependent manner (Fig. 3a).

Inflammatory chemokines comprise a family of small (6–14 kDa) structurally related proteins that play major roles in the activation, differentiation, and infiltration of various immune cells in AD lesions^42^. Among these chemokines, thymus- and activation-regulated chemokine (TARC), macrophage-derived chemokine (MDC), regulated upon activation normal T cell expressed and secreted (RANTES), and eotaxin are members of the C–C chemokine family and affect the regulation of immune cells during AD progression^43^. MDC and TARC are ligands of C–C chemokine receptor type (CCR)4, which regulates the activation of Th2 cells critical for allergic reactions^44^. RANTES and eotaxin are CCR3 ligands that play an active role in regulating the infiltration and activation of immune cells, such as mast cells, basophils, and eosinophils, in AD lesions^44^. Therefore, inhibiting the secretion of inflammatory chemokines, such as TARC, MDC, RANTES, and eotaxin, is expected to suppress AD-related inflammatory responses. To confirm the inhibitory effects of SHE on chemokine levels in DFE-induced *NC/Nga* mouse blood serum, we measured these using a bead-based immunoassay. The increase in the DFE-induced TARC, MDC, RANTES, and eotaxin levels in AD mouse serum was significantly suppressed by oral SHE administration (Fig. 3b). In particular, the serum levels of TARC and MDC, crucial chemoattractants of T cells, are considered biomarkers for assessing the severity of AD in adults. Numerous non-clinical and clinical studies have provided substantial evidence of a strong correlation with the clinical severity of AD, both at baseline and throughout therapy^45,46^. To elucidate this intricate relationship, we quantified TARC, MDC, RANTES, and eotaxin protein levels in skin tissues subjected to induced AD. The serum-derived results for RANTES and eotaxin exhibited consistency with those ascertained from the skin tissue. In contrast, the surge in TARC protein concentrations in the skin tissue after induction did not correspond proportionately to the augmented serum levels. For MDC in skin tissue, a noticeable increase after induction was observed. However, no decrease was detected after SHE administration (data not shown).

These findings suggest that while TARC and MDC can serve as effective serum biomarkers, disparities exist in certain research findings where serum outcomes did not consistently mirror the results from skin tissue analysis in AD, possibly depending on the pathological state of the lesion. Moreover, despite the observed differences between skin and blood biomarkers, the substantial association between serum cytokines and disease activity, as delineated by SCORAD, underscores their potential clinical significance. This contrast between serum and lesional skin biomarkers accentuates that circulating cytokine profiles could encapsulate a broader immune activation. This underscores the imperative of deciphering immune mechanisms beyond cutaneous lesions, particularly regarding AD-associated comorbidities and inherent risks^47–49^. The DFE-induced NC/Nga mouse model is designed to reflect both the acute and chronic stages of AD, encompassing a combination of Th1 and Th2 responses^50^. In the acute phase, the Th2 response predominates, with increased levels of Th2 cytokines, while the chronic stage is characterized by a Th1/Th2 mixed response, with the activation of Th1 cytokines such as IFN-γ becoming more evident. This representation aligns with clinical observations of human AD, where enhanced Th1 responses are often seen at chronic stages^50,51^. Therefore, we confirmed the serum levels of Th1 cytokines, such as IFN-γ and TNF-α serum levels, in DFE-treated *NC/Nga* mice. Increased IFN-γ and TNF-α serum levels were suppressed in mice following SHE administration (Fig. 3c). These results suggested that SHE exerts an anti-atopic effect by inhibiting the release of inflammatory mediators in DFE-treated *NC/Nga* mice.

### Inhibitory effects of SHE on IFN-γ/TNF-α-induced inflammatory chemokine production in HaCaT cells

**Figure 4:**
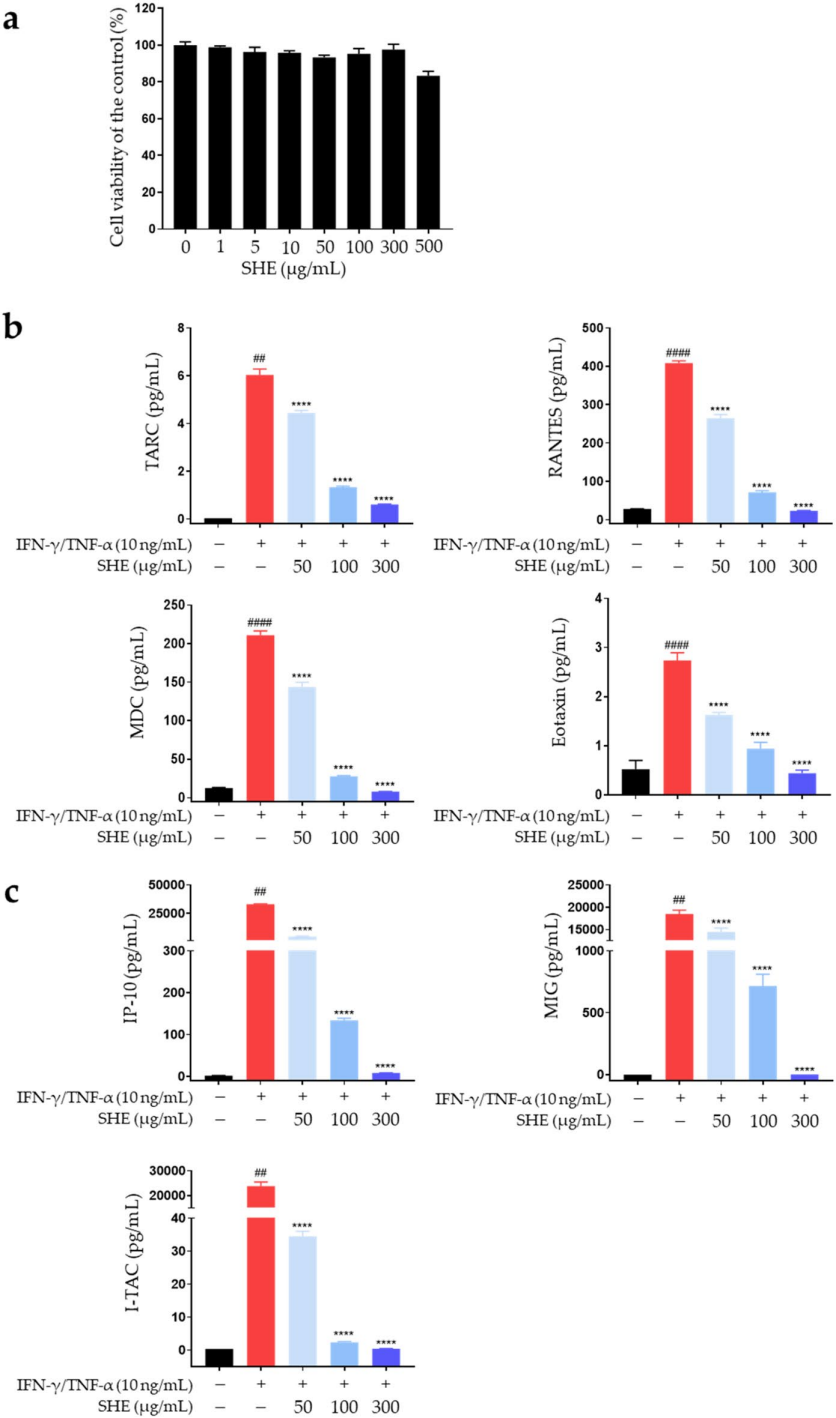
SHE inhibits the secretion of inflammatory chemokines in IFN-γ/TNF-α-stimulated HaCaT cells. (**a**) Cytotoxicity of SHE was determined after treatment (0–500 μg/mL) for 24 h. (**b**, **c**) Secreted MDC levels were measured using ELISA. The levels of TARC, RANTES, eotaxin, IP-10, MIG, and I-TAC were determined using a bead-based immunoassay. The cells were pre-treated with SHE for 1 h and then incubated with IFN-γ/TNF-α for 24 h. Results are presented as mean ± SEM (n = 3). #*P* < 0.05, ##*P* < 0.005, ###*P* < 0.0005, ####*P* < 0.0001 vs. untreated; *P < 0.05, **P < 0.005, ***P < 0.0005, ****P < 0.0001 vs. IFN-γ/TNF-α.

Epidermal keratinocytes in AD-affected skin are exposed to various stimuli and play a critical role in immune responses through the production of inflammatory mediators, including chemokines^44^. TARC, RANTES, MDC, and eotaxin, among others, are secreted from keratinocytes following IFN-γ/TNF-α co-stimulation^52^. Before confirming the inhibitory effects of SHE on chemokine release, we confirmed its cytotoxicity by exposing HaCaT cells to various concentrations (0–500 μg/mL) for 24 h to determine the optimal concentration for our experiment. SHE had no toxic effects on cell viability up to a concentration of 500 µg/mL (Fig. 4a). Therefore, the cells were treated with 50–300 µg/mL SHE in subsequent experiments. To confirm the inhibitory effects of SHE on chemokine production, HaCaT cells were pre-treated with SHE for 1 h followed by IFN-γ/TNF-α treatment for 24 h. The supernatant was then collected to measure chemokine levels. SHE suppressed the IFN-γ/TNF-α-induced production of TARC, RANTES, MDC, and eotaxin in HaCaT cells (Fig. 4b).

C–C chemokines play important roles in the activation and infiltration of Th2 cells in patients with acute AD^43^. In general, the pathogenesis of acute AD is characterized by Th2-dominant inflammation, highlighting the importance of controlling Th2 responses during AD treatment^40^. However, Th1-mediated responses are more prominent in patients with chronic AD, with high IFN-γ secretion by Th1 cells playing a crucial role in the chronic AD response^5,53^. Th1 activation depends on IFN-γ-induced C-X-C chemokines such as IP-10, MIG, and I-TAC, which are secreted from the activated keratinocytes within AD skin lesions^53,54^. Their interactions with C-X-C chemokine receptor type (CXCR) 3 primarily occur on activated Th1 cells rather than on Th2 cells^44,53,55^. Therefore, we determined the effects of SHE on IFN-γ/TNF-α-induced C-X-C chemokine (IP-10, MIG, and I-TAC) secretion in HaCaT cells. IFN-γ/TNF-α treatment significantly induced IP-10, MIG, and I-TAC secretion, which was suppressed by SHE treatment (Fig. 4c). These results suggest that SHE inhibits the release of several chemokines, such as TARC, RANTES, MDC, eotaxin, IP-10, MIG, and I-TAC, by keratinocytes within AD skin lesions. This regulation by SHE indicates its potential to inhibit AD-associated inflammation.

### Inhibitory effects of SHE on IFN-γ/TNF-α-induced mitogen-activated protein kinase (MAPK)/activator of transcription 1 (STAT1) signaling in HaCaT Cells

**Figure 5:**
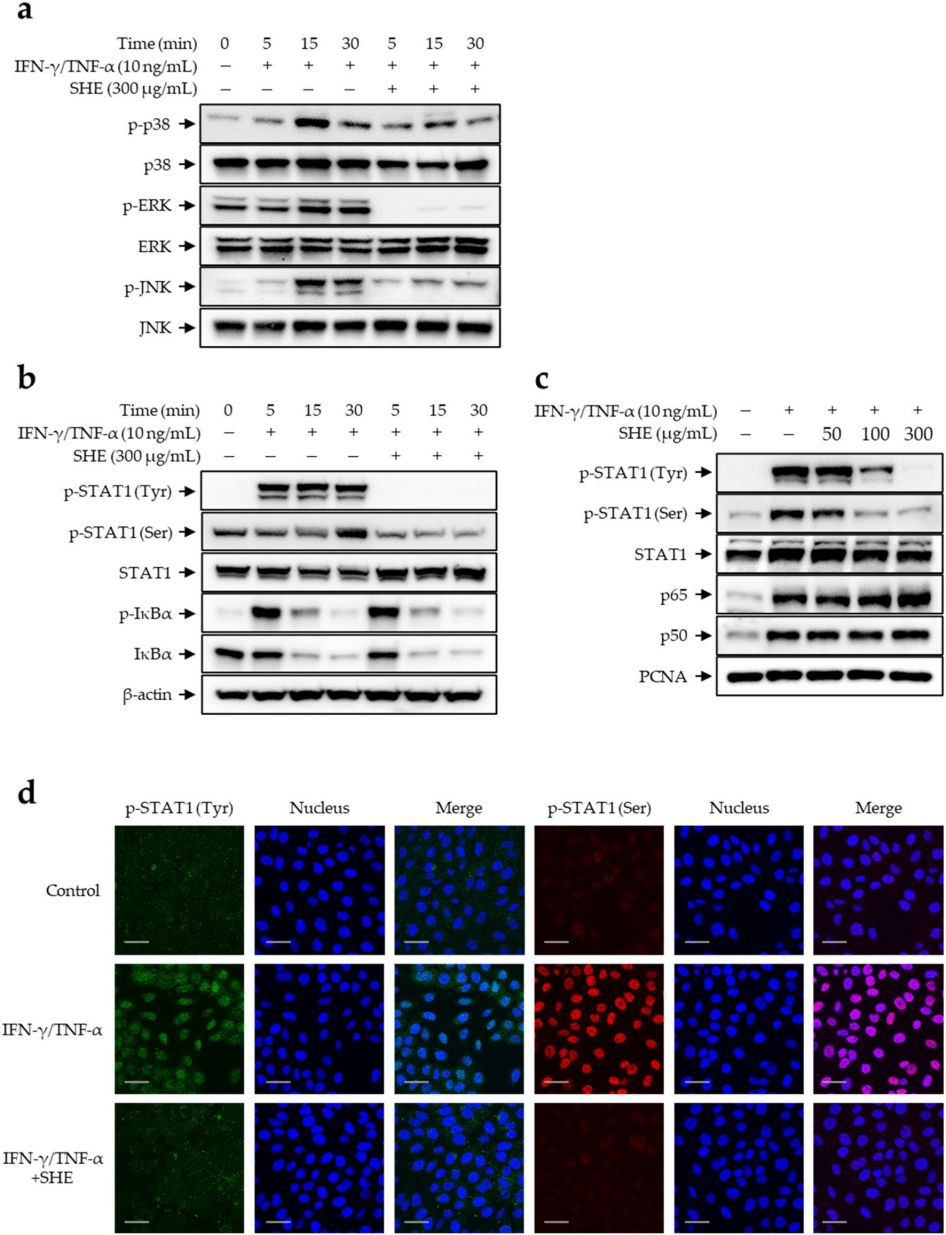
SHE inhibits the MAPK/NF-κB/STAT1 signaling pathway in IFN-γ/TNF-α-stimulated HaCaT cells. The cells were pre-treated with SHE (300 µg/mL) for 1 h and then stimulated with IFN-γ/TNF-α for 0, 5, 15, and 30 min. (**a**) Phosphorylated and total MAPK (p38, ERK, and JNK) protein levels as well as (**b**) phosphorylation or degradation of IκBα and STAT1 were detected in the cells. (**c**, **d**) HaCaT cells were pre-stimulated with SHE for 1 h and then treated with IFN-γ/TNF-α for 1 h. (**c**) Nuclear extracts were analyzed using western blotting to detect p-STAT1, STAT1, and NF-κB (p65 and p50 subunits). PCNA was used as a loading control for nuclear extracts. For clarity of data, the Western blots were cropped and all original blots are presented in Supplementary Figure 1. (**d**) Localization of p-STAT1 (green and red) was visualized using immunofluorescence. The nuclei were stained with DRAQ (blue). Scale bar = 10 μm.

Atopic inflammatory reactions are regulated through several intracellular pathways, including MAPK signaling, as shown in both AD mice and keratinocytes^15,56,57^. IFN-γ/TNF-α co-stimulation increases the phosphorylation and activation of p38, extracellular signal-regulated kinases (ERK), as well as c-Jun N-terminal kinases (JNK) in HaCaT cells^57^. In keratinocytes, the MAPK cascade drives the production of chemokines, such as TARC, RANTES, eotaxin, IP-10, MIG, and I-TAC, through the regulation of several transcriptional factors, including nuclear factor kappa B (NF-κB) and STAT1^54,57,58^. STAT1 is phosphorylated following stimulation with IFN-γ/TNF-α, translocated to the nucleus, and binds to target gene promoters, activating expression. Similarly, NF-κB activation via inhibitor kappa B-alpha (IκB-α) phosphorylation leads to the degradation of IκB-α, causing the translocation of NF-κB into the nucleus, where it activates target inflammatory gene expression by binding to regulatory elements within the promoter^59^. Next, we determined the regulatory effect of SHE on the IFN-γ/TNF-α-induced phosphorylation of MAPKs (p38, ERK, and JNK) and transcriptional factors (NF-κB and STAT1) in HaCaT cells. SHE treatment suppressed the phosphorylation of MAPKs in IFN-γ/TNF-α-stimulated HaCaT cells (Fig. 5a). In addition, the increased phosphorylation of STAT1 (Tyr) and STAT1 (Ser) induced by IFN-γ/TNF-α stimulation in HaCaT cells was suppressed via SHE pre-treatment (Fig. 5b, upper panel). However, SHE did not inhibit the phosphorylation and degradation of IFN-γ/TNF-α-induced IκB-α (Fig. 5b, lower panel). Furthermore, to determine the effects of SHE on STAT1 (Tyr and Ser) and NF-κB (p65 and p50) nuclear translocation, the nuclear fraction of stimulated cells was analyzed via western blotting. Consistent with the results in Fig. 5c, IFN-γ/TNF-α-induced phospho-STAT1 translocation was markedly reduced by SHE treatment, whereas the translocation of NF-κB subunits p65 and p50 was affected. Immunofluorescence staining confirmed the IFN-γ/TNF-α-induced nuclear translocation of phospho-STAT1 (Fig. 5d). These results suggest that SHE exerts anti-inflammatory effects by blocking MAPK/STAT1 activation.

### High-performance liquid chromatography analysis of two reference compounds in SHE

**Figure 6:**
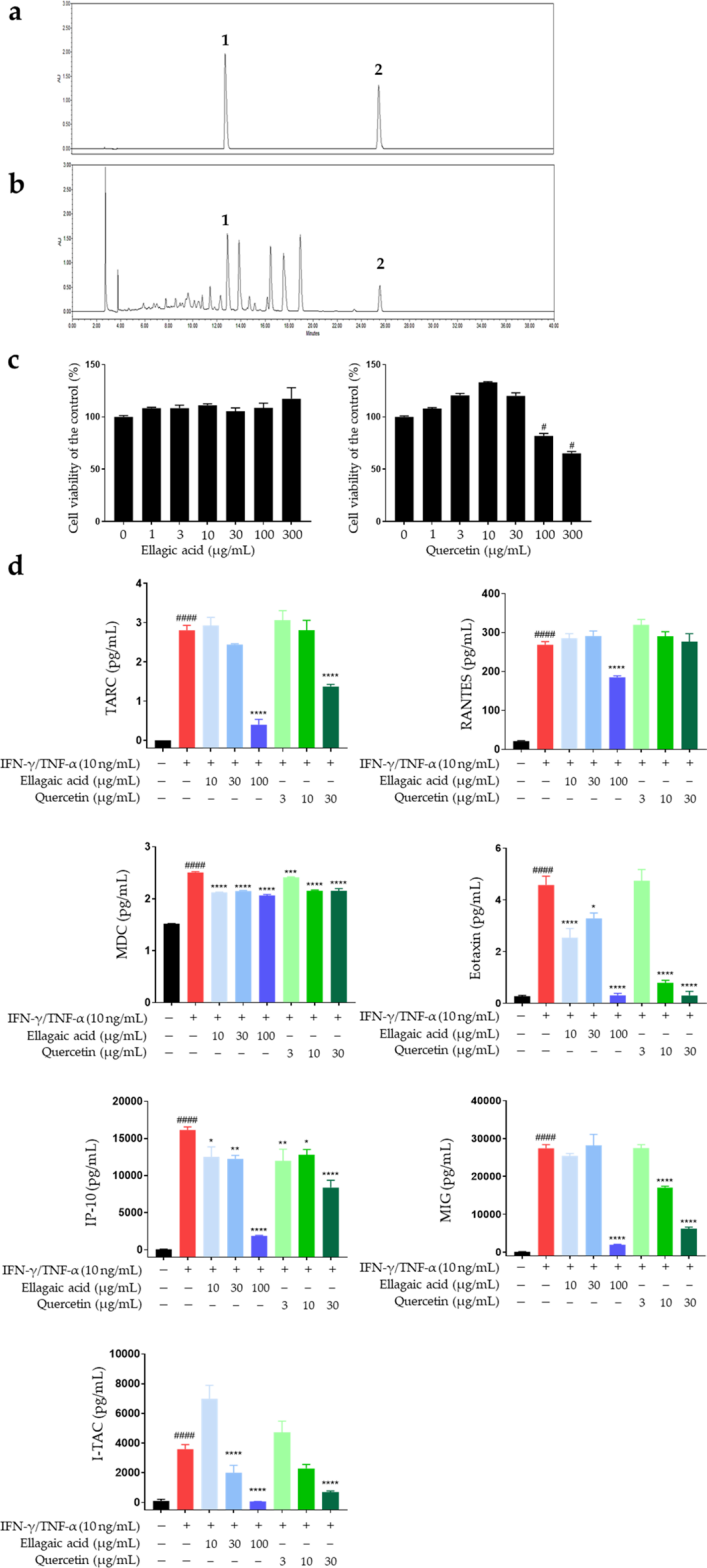
Two compounds from *Sanguisorba hakusanensis* ethanol extract (SHE) inhibit the secretion of inflammatory chemokines in IFN-γ/TNF-α-stimulated HaCaT cells. HPLC chromatograms of the standard mixture solution (**a**) and SHE (**b**). The identified compounds are ellagic acid (1) and quercetin (2). (**c**) Cytotoxicity of the compounds was analyzed after treatment (0–300 μg/mL) for 24 h. (**d**) Secreted macrophage-derived chemokine (MDC) levels were measured using ELISA. The levels of TARC, RANTES, eotaxin, IP-10, MIG, and I-TAC were determined using a bead-based immunoassay. The cells were pre-treated with two compounds for 1 h and then incubated with IFN-γ/TNF-α for 24 h. Results are presented as mean ± SEM (n = 3). #*P* < 0.05, ##*P* < 0.005, ###*P* < 0.0005, ####*P* < 0.0001 vs. untreated; *P < 0.05, **P < 0.005, ***P < 0.0005, ****P < 0.0001 vs. IFN-γ/TNF-α.

Two reference compounds in SHE were simultaneously identified via high-performance liquid chromatography (HPLC) analysis. A representative HPLC chromatogram of the standards and SHE at a detection wavelength of 250 nm is shown in Fig. 6a,b. The two components detected were ellagic acid and quercetin, with retention times of 12.76 and 25.53 min, respectively. Table 1 shows the linearity, limit of detection (LOD), and limit of quantitation (LOQ). The correlation coefficients (r^2^) of the calibration curves for the two compounds were ≥ 0.9998, indicating good linearity. The LOD and LOQ were in the range of 0.01–0.02 and 0.03–0.05 µg/mL, respectively. The ellagic acid and quercetin content in the samples were 5.989 and 4.952 mg/g, respectively (Table 2).

**Table 1:** Linear regression data, LOD, and LOQ of ellagic acid and quercetin.

Compound	Calibration curve^a^	Correlation (R^2^)	Linear range (µg/mL)	LOD^b^ (µg/mL)	LOQ^c^ (µg/mL)
Ellagic acid	y = 27,198–x–242,107	0.9998	2.50–160.00	0.02	0.05
Quercetin	y = 11,445–x–113,872	0.9999	2.19–140.00	0.01	0.03

^a^y = peak area; x = concentration of the reference compound (µg/mL). ^b^LOD (limit of detection) = 3.3 × SD / S. ^c^ LOQ (limit of quantification) = 10 × SD / S (SD: standard deviation of the blanks, S: slope of the calibration curve).

**Table 2:** Content of the two compounds in SHE.

Compound	Content (mg/g)		
	Mean	SD	RSD (%)
Ellagic acid	5.989	0.001	0.025
Quercetin	4.952	0.008	0.170

To determine suitable experimental concentrations of ellagic acid and quercetin, we first assessed their cytotoxicity (Fig. 6c). Therefore, the cells were treated with 10–100 µg/mL ellagic acid and 3–30 µg/mL quercetin in subsequent experiments. The two compounds suppressed the IFN-γ/TNF-α-induced production of TARC, RANTES, MDC, eotaxin, IP-10, MIG, and I-TAC in HaCaT cells (Fig. 6d). These results showed that the reference compounds of SHE exert synergistic anti-AD effects.

Ellagic acid is a natural polyphenol antioxidant found in several fruits, vegetables, and nuts^60^. It has a variety of beneficial properties, including hepatoprotective, anti-cancer, anti-diabetes, and anti-inflammatory properties^60^. Notably, the anti-allergic effects of ellagic acid have been reported in acute or chronic allergy disease models, including AD and asthma models^61–63^. Quercetin is another naturally produced polyphenol flavonoid, found in certain fruits and vegetables, including apples, berries, tomatoes, grapes, onions, and shallots^64,65^. It has anti-cancer, anti-inflammatory, and anti-atherosclerotic activities^66,67^. Quercetin also exerts inhibitory effects on several allergic reactions, including AD^65^. In IFN-γ/TNF-α-stimulated HaCaT cells, the anti-atopic effect of ellagic acid involves the MAPK/STAT pathway, whereas that of quercetin involves the MAPK/NF-κB pathway^63,68,69^. Therefore, the AD-inhibitory effects of SHE observed in this study could be attributed to the influence of ellagic acid. Despite attempts at chemical profiling of SHE, only two components have been identified in our HPLC analysis. Therefore, it is necessary to perform additional chemical profiling of SHE through liquid chromatography mass spectrometry (LC–MS) or gas chromatography analysis to identify the major active components with anti-AD effects through bioactive-guided isolation.

In conclusion, we found that the administration of SHE reduced AD-like symptoms, such as epidermal hyperplasia, itching, and mast cell infiltration, in *NC/Nga* mice with DFE-induced AD. In addition, SHE suppressed the serum levels of IgE, histamine, and Th2-related inflammatory chemokines. Furthermore, it regulated the expression of inflammatory chemokines in IFN-γ/TNF-α-stimulated HaCaT cells via the MAPK/STAT1 pathway. Although further research is required to characterize the inhibitory effects of SHE on Th1-related inflammatory responses in chronic AD, our results suggest that SHE represents an effective alternative therapeutic agent for AD. In addition, although further comparisons of efficacies with other *Sanguisorba* species are needed, the discovery of previously unreported medicinal properties in this novel plant suggests the potential to identify an even greater diversity of medicinal plants within the genus *Sanguisorba*.

## Methods

### Reagents

IFN-γ and TNF-α were purchased from Thermo Fisher Scientific (Waltham, MA, USA). High-glucose-containing Dulbecco’s modified Eagle’s medium (DMEM) was purchased from Hyclone (Logan, UT, USA). Phosphate-buffered saline (PBS), fetal bovine serum (FBS), and penicillin/streptomycin were obtained from Gibco-BRL (Gaithersburg, MD, USA). DMSO, sodium dodecyl sulfate (SDS), avertin, and dexamethasone were purchased from Sigma-Aldrich (St. Louis, MO, USA). Phospho (p)-STAT1 (Tyr), p-STAT1 (Ser), STAT1, p-IκBα, IκBα, p-ERK, ERK, p-p38, p38, p-JNK, JNK, p65, and β-actin antibodies were obtained from Cell Signaling Technology (Beverly, MA, USA). PCNA, p50, and secondary antibodies were purchased from Santa Cruz Biotechnology (Santa Cruz, CA, USA).

### SHE preparation

The roots of *Sanguisorba hakusanensis* Makino were harvested in July 2018 from wild populations in the Jeollabuk-do region of South Korea, specifically within the Deogyu-mountain area. The specimens were authenticated by Professor Byung-Kil Choo from the Department of Crop Science & Biotechnology at Jeonbuk National University. After collection, the samples were thoroughly cleaned and air-dried at ambient temperature. Upon complete desiccation, the roots were pulverized into a fine powder and preserved at − 70 °C for subsequent analyses. The plant was extracted three times for 2 h at 50 ± 2 °C with 70% ethanol at a ratio of 1:10. SHE was then filtered, lyophilized, and concentrated using a rotary vacuum evaporator under a reduced pressure to obtain crude extracts. Finally, SHE powder was freeze-dried at − 88 °C and stored until further use at − 20 °C. For the in vivo study, SHE powder was dissolved in water for oral gavage. For the in vitro study, the powder extract was dissolved in dimethyl sulfoxide (DMSO) solution and sterilized using a 0.22-μm syringe filter. The plant name has been checked with “The Plant List” website; (www.theplantlist.org).

### Animals and induction of atopic skin inflammation

Eight-week-old male *NC/Nga* mice were purchased from Central Lab Animal Inc. (Seoul, Korea). The mice were individually housed under controlled conditions and maintained under specific pathogen-free conditions for 1 week before the experiment. For the experimental set up, mice were randomly divided into five groups (6 mice/group). To disrupt the skin barrier, 150 μL of 4% SDS was sprayed on the dorsal skin and ear of the mice, which were shaven the previous day. After 3 h, 100 mg of DFE (Biostir Inc., Kobe, Japan) was applied to the same area to induce AD-like skin lesions. AD was induced twice a week for a total of 3 weeks. From day 9 of the experiment, SHE and dexamethasone (acting as the positive control drug) dissolved in water were orally administered daily for 14 days (Fig. 1a). All experimental procedures were performed according to the guidelines of the Institutional Animal Care and Use Committee at the Korea Institute of Oriental Medicine (KIOM) under certification number 21–061. This study is reported in accordance with ARRIVE guidelines.

### Measurement of dermatitis score, ear thickness, and behavior

Clinical dermatitis score, ear thickness, and scratching behavior were evaluated twice a week. According to the scoring system^70^, the total dermatitis score was the sum of individual scores: 0 (no symptoms), 1 (mild), 2 (moderate), or 3 (severe) based on the presence of AD symptoms (edema, scarring/dryness, erythema/hemorrhage, and excoriation/erosion) in the skin and ear lesions. As a parameter of cutaneous inflammation, ear thickness was measured in the same site using a digital caliper (CAS, Seoul, Korea). Scratching behavior was assessed in each mouse group. The mice were placed in a transparent plastic cage and observed for 20 min to evaluate scratching of the ears, face, or back using their hind paws^71^.

### Serum analysis

For serum collection in experimental animals, anesthetization was performed using 240 mg/kg of avertin via intraperitoneal injection, and the animal was sacrificed by cervical dislocation to minimize pain. Blood was centrifuged to obtain serum, which was then stored at − 80 °C until analysis. Total serum IgE level was determined using the LBIS Mouse IgE ELISA Kit (Fujifilm, Shibukawa, Japan), and serum histamine levels were determined using Histamine Research ELISA™ (LDN, Nordhorn, Germany). The total serum levels of TARC, MDC, RANTES, eotaxin, IFN-γ, and TNF-α were determined using the LEGENDplex™ Mouse Proinflammatory Chemokine Panel and LEGENDplex™ Mouse Inflammation Panel 1 (BioLegend, San Diego, CA, USA) bead‐based immunoassays. The samples were measured using the BD LSRFortessa Flow Cytometer (BD Biosciences, San Jose, CA, USA) with BD CellQuest™ software. Data were analyzed using LEGENDplex™ Software v8.0 (VigeneTech Inc., Carlisle, MA, USA). All experiments were conducted according to the manufacturer’s protocol.

### Histopathological observation

The mouse tissue samples were fixed in 10% formalin. For tissue staining, the samples were embedded in paraffin and cut into 4-μm-thick sections. The sections were stained with hematoxylin/eosin solution (H&E) (Sigma-Aldrich, St. Louis, MO, USA) or Toluidine Blue (TB) O solution (Sigma-Aldrich). For immunohistochemistry, the sections were incubated overnight at 4 °C with F4/80 antibodies and processed using an XT System BenchMark autostainer (Ventana Medical System, Tucson, AZ, USA) according to the manufacturer’s instructions. The stained tissue samples were observed using a digital slide scanner (Kfbio, Ningbo, China). The tissue sections were analyzed using Solution for Automatic Bio-Image Analysis software (EBIOGEN, Seoul, Korea).

### Cell culture and assessment of cell viability

HaCaT cells were obtained from Elabscience (Houston, TX, USA). The cells were cultured in high-glucose DMEM containing 10% heat-inactivated FBS and 1% penicillin/streptomycin, then incubated in a 5% CO_2_ incubator at 37 °C. To assess cell viability, HaCaT cells seeded in each well of a 96-well plate were treated with SHE (0–500 μg/mL) or left untreated for 24 h. Pre-warmed 20 μL of Cell Counting Kit-8 (Dojindo Molecular Technologies Inc., Rockville, MD, USA) reagent was added to each well, and the plate was incubated in a humidified 5% CO_2_ chamber at 37 °C for 2 h. The optical density of these mixtures was measured using a microplate reader (SpectraMax 340; Molecular Devices, CA, USA) at 450 nm.

### Inflammatory chemokine analysis

HaCaT cells were pre-stimulated with SHE for 1 h and then incubated with 10 ng/mL IFN-γ/TNF-α for 24 h. The culture medium was then harvested, and the release of TARC, RANTES, eotaxin, IFN-γ-induced protein 10 (IP-10), monokine induced by IFN-γ (MIG), and IFN-inducible T cell-chemoattractant (I-TAC) in the supernatant was confirmed using the LEGENDplex™ Proinflammatory Chemokine panel (BioLegend, San Diego, CA, USA) bead‐based immunoassay. Prepared samples were analyzed using the BD LSRFortessa Flow Cytometer (BD Biosciences) with BD CellQuest™ software. Data were evaluated using LEGENDplex™ software v8.0 (VigeneTech Inc.). Secreted MDC protein in the supernatants was measured using Human CCL22/MDC Quantikine ELISA Kit (R&D Systems, Minneapolis, MN, USA). The absorbance was detected using a microplate reader (Molecular Devices). All experimental processes were performed according to the manufacturer’s instructions.

### Western blotting

The cells were pre-stimulated with 300 μg/mL SHE for 1 h and then treated with 10 ng/mL IFN-γ and 10 ng/mL TNF-α at each experimental time point at 37 °C. To analyze the total cell lysates, the cells were lysed with PRO-PREP extraction buffer (Intron, Seoul, Korea) for 30 min on ice, and the lysates were centrifuged at 13 000 rpm for 10 min at 4 °C. To analyze the nuclear proteins, the cells were prepared using NE-PER® Nuclear and Cytoplasmic Extraction Reagents (Pierce Biotechnology, Rockford, IL, USA) containing Halt Protease and Phosphatase Inhibitor Cocktail (Thermo Fisher Scientific). Protein concentrations were then determined using the Pierce™ BCA assay kit (Thermo Fisher Scientific). Equal amounts of the extracted proteins (10 μg) were separated using electrophoresis with a 10% Mini-PROTEAN TGX Precast Protein Gel (Bio-Rad, Hercules, CA, USA) and electrophoretically transferred onto Hybond™ polyvinylidene fluoride membranes (GE Healthcare Life Sciences, Little Chalfont, UK) using a western blot apparatus. Each membrane was blocked with 5% skim milk (Sigma-Aldrich, St. Louis, MO, USA) or 5% bovine serum albumin (BSA; MP Biomedicals, Irvine, CA, USA) in TBS with Tween-20 at 4 °C for 2 h. Primary antibodies (1:1000 dilution) were diluted in blocking solution, and the membranes were incubated with the antibody solutions at 4 °C overnight. Secondary antibodies (horseradish peroxidase-conjugated anti-IgG, 1:2500) in blocking solution were added on the membranes after washing the primary antibodies and incubated for 1 h. The stained proteins were developed using Super Signal West Femto Chemiluminescent Substrate (Thermo Fisher Scientific). The ChemiDoc Imaging System (Bio-Rad) was used to visualize protein expression, which was quantified using Image Lab software v5.2.1 (Bio-Rad). Data are expressed as mean ± SEM (n ≥ 3).

### HPLC analysis

HPLC analysis was performed using a Waters e2695 liquid chromatography system (Waters Co., Milford, MA, USA) equipped with a Waters 2998 photodiode array detector. All analyses were performed using Empower software v3 (Waters Co., Milford, MA, USA). Chromatographic separation was conducted on a Waters Xselect C18 column (250 mm × 4.6 mm; 5 μm, Milford). An elution system with 0.1% aqueous trifluoroacetic acid (A) and acetonitrile (B) was used at the following gradients: 0–5 min, 10%–20% B; 5–40 min, 20%–45% B. After each analysis, the equilibration time used to return to the initial mobile phase composition (10% B) was 8 min. The flow rate of the mobile phase was 1.0 mL/min, and the injection volume was 20 µL. The detection wavelength selected for quantitative analysis of the samples was 250 nm. SHE was dissolved in methanol (10 mg/mL) and passed through 0.2 μm syringe filters before HPLC analysis. Two reference compounds with > 98% purity were used for the analysis; ellagic acid was purchased from ChemFaces Biochemical (Wuhan, China), and quercetin was purchased from Biopurify Phytochemicals (Chengdu, China).

### Immunofluorescence staining

HaCaT cells (6 × 10^5^ cells/mL) were grown in a 12-mm Nunc Glass Base dish (Thermo Fisher Scientific) for 24 h. The cells were then pre-stimulated with SHE for 1 h and incubated with IFN-γ and TNF-α (10 ng/mL each) for 1 h. The cells were then fixed with 3% formaldehyde dissolved in PBS for 20 min. After being washed with 0.1% TritonX-100 (LPS solution, Daejeon, Korea) in PBS (PBST), the cells were blocked and permeabilized using 3% BSA buffer diluted with PBST for 1 h, then incubated overnight at 4 °C with the same primary antibodies (1:500) as those used for western blot analysis, with 3% BSA in PBST. The cells were washed three times with PBST and stained with Alexa Fluor 594 or 488 anti-rabbit IgG secondary antibody solutions (1:500 dilutions; Thermo Fisher Scientific) for 2 h in the dark at 4 °C. The nuclei were labeled with DRAQ in blocking buffer for 15 min. After being washed four times with PBST, the cells were observed under a FV10i confocal microscope (Olympus, Tokyo, Japan).

### Statistical analysis

All experiments were performed independently at least three times. GraphPad Prism v8.0 (GraphPad Software, San Diego, CA, USA) was used to conduct statistical analyses. Data are shown as the mean ± SEM and subjected to analysis of variance (ordinary one-way ANOVA). Differences between groups were considered statistically significant at *P* < 0.05.

### Ethical approval

All experiments and methods were approved and performed in accordance with relevant guidelines and regulations of KIOM.

### Supplementary Information

Supplementary Figure 1.

## Data Availability

The data presented in this study are available on request from the corresponding author.
