# Supplementary figures and images for: Inhibitory effect of Sanguisorba hakusanensis Makino ethanol extract on atopic dermatitis-like responses in NC/Nga mice and human keratinocytes
Source: Sci Rep. 2023 Sep 5;13:14594. doi: 10.1038/s41598-023-41676-3 (PMC10480230; doi:10.1038/s41598-023-41676-3)

**Figure 5a**

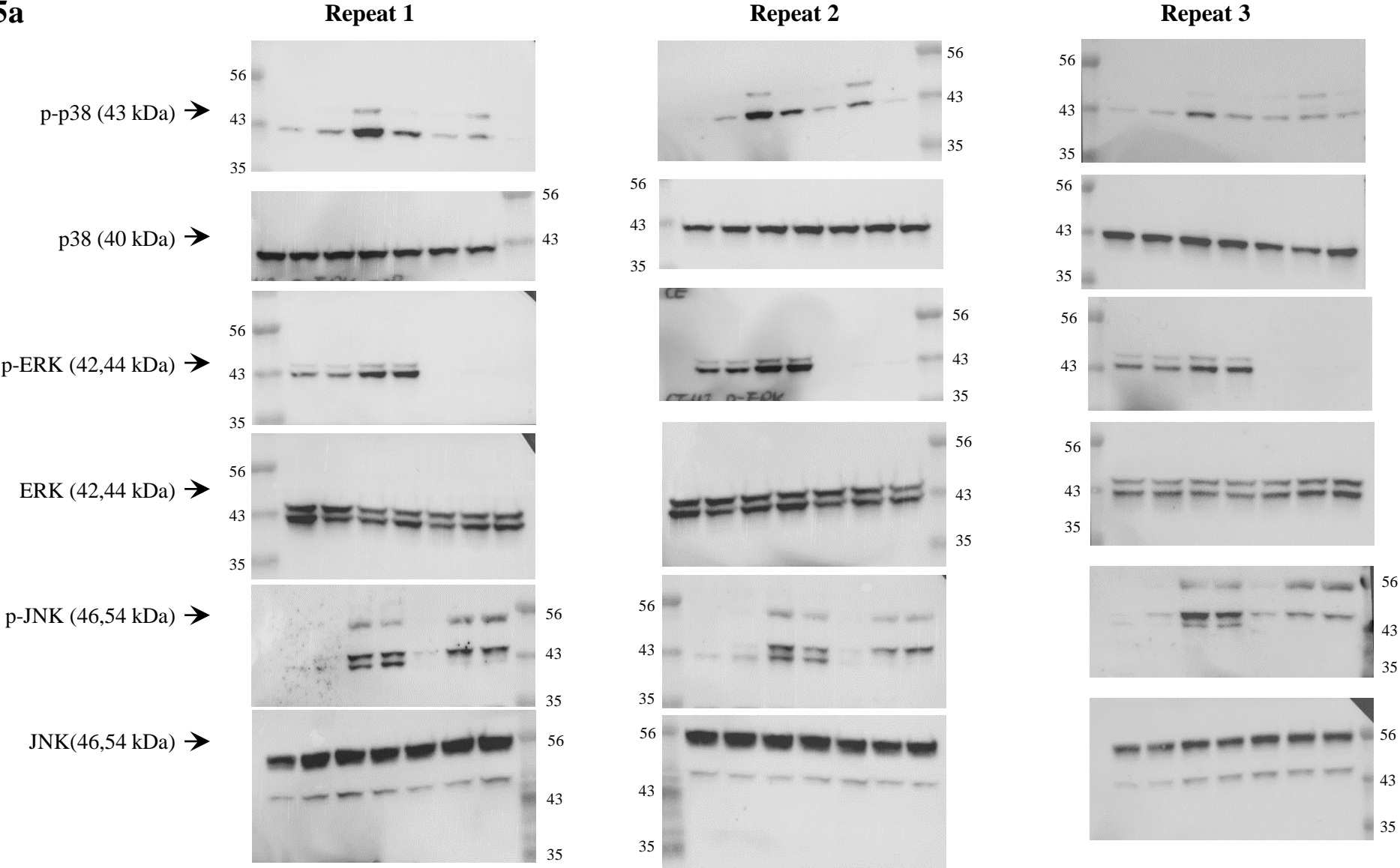

Figure 5b

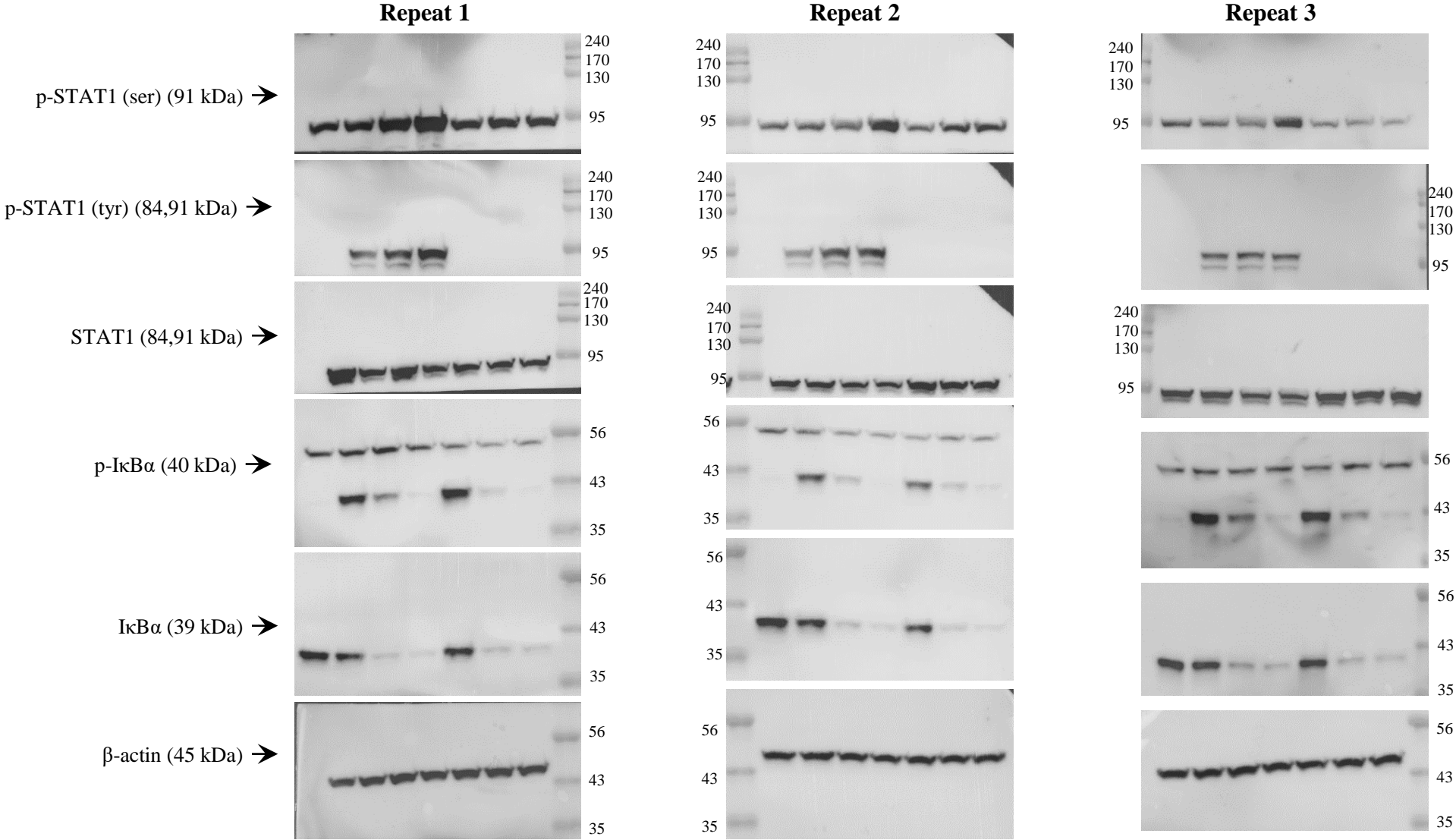

**Figure 5c**

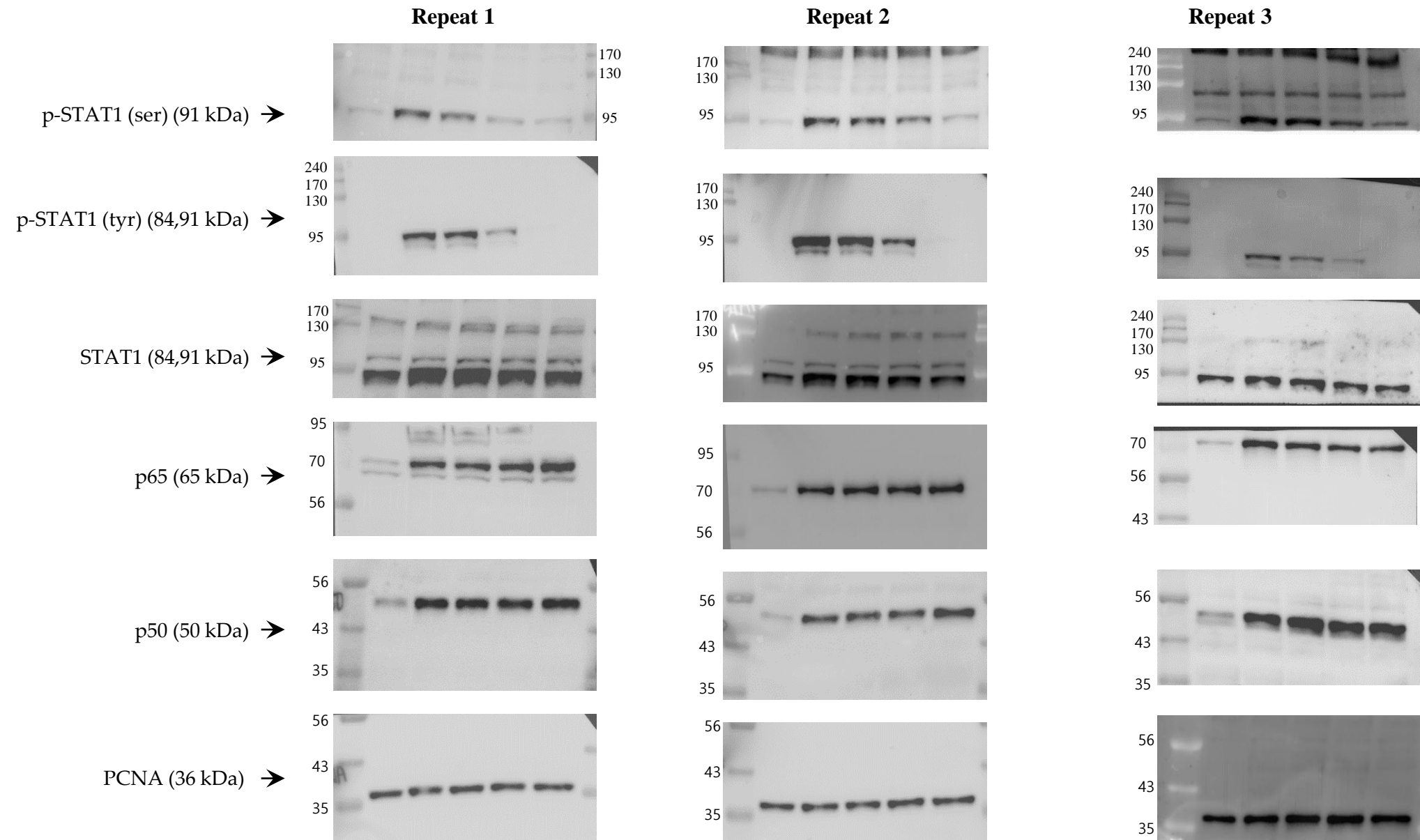

Supplement: Supplementary file 1 — Supplementary Figure 1. [file 41598_2023_41676_MOESM1_ESM.pdf]
